# Ease of Handling and Physiological Parameters of Stress, Carcasses, and Pork Quality of Pigs Handled in Different Group Sizes

**DOI:** 10.3390/ani9100798

**Published:** 2019-10-14

**Authors:** Filipe Antonio Dalla Costa, Osmar Antonio Dalla Costa, Izabela Cruvinel Di Castro, Neville George Gregory, Melissa Selaysim Di Campos, Guilherme Brunno de Medeiros Leal, Fernando de Castro Tavernari

**Affiliations:** 1 Programa de Pós-graduação em Zootecnia, Faculdade de Ciências Agrárias e Veterinárias, Universidade Estadual Paulista, UNESP-FCAV, Jaboticabal 14884-900, Brazil; 2 Embrapa Suínos e Aves (Embrapa Swine and Poultry), BR 153, Km 110, Concórdia 89700-991, Brazil; osmar.dallacosta@embrapa.br (O.A.D.C.); fernando.tavernari@embrapa.br (F.d.C.T.); 3 Programa de Pós-graduação em Zootecnia, Escola de Veterinária e Zootecnia, Universidade Federal de Goiás, Goiânia—GO 74.045-155, Brazil; izabelazootecnia@gmail.com (I.C.D.C.); melissa@ufg.br (M.S.D.C.); gmzootecnia@gmail.com (G.B.d.M.L.); 4 Royal Veterinary College, University of London, Hatfield E16 2PX, UK; neville@fcav.unesp.br

**Keywords:** cortisol, lactate, mounting, pH, skin lesion

## Abstract

**Simple Summary:**

The effect of different group sizes of pigs (3, 5, and 10 pigs) during handling on physiological parameters, carcasses, and pork quality traits at the farm and slaughterhouse were evaluated in 360 pigs. Ease of handling decreased as the group size increased. Moving pigs in smaller groups improve animal welfare parameters and carcass quality. However, meat quality classifications of the carcasses were not affected by the groups size used in preslaughter handling. Based on the results, moving groups of five pigs seems to be the best strategy to improve animal welfare and carcass quality.

**Abstract:**

The effect of different group sizes of pigs (3, 5, and 10 pigs) during handling on physiological parameters, carcasses, and pork quality traits at the farm and slaughterhouse were evaluated in 360 pigs from five farms (four repetitions or group/treatment/farms). Data was analyzed as a factorial of 3 × 5 (3 treatments × 5 farms) to check effects of treatments by analysis of variance in ANOVA. Ease of handling decreased as the group size increased. However, time taken in handling was not influenced by the group size (*p* > 0.10). Moving pigs in groups of five animals reduced effects on blood cortisol levels (*p* < 0.05). Fighting and handling lesions in the carcasses increased for bigger handling groups (*p* < 0.05). Pigs handled in groups of three and ten animals had a higher pHu and initial temperature in Longissimus thoracis and Semimembranosus (*p* < 0.05) and lower drip loss in Semimembranosus (*p* < 0.05). However, meat quality classifications of the carcasses were not affected by treatments. Based on the results, moving groups of five pigs seems to be the best strategy to improve animal welfare, carcasses and pork quality.

## 1. Introduction

Animals need to be moved during the production cycle between facilities, pens, and alleys for vaccination, weighing, and transport (loading/unloading procedures) [1]. The handling procedures adopted can impact the pigs’ responses to stress, their welfare, the ease of handling, time spent in handling, and the staff workload.

For example, a proper handling during loading to transport significantly increases the heart rate and blood pressure [2]. However, when improperly performed, pigs are physically challenged [3] by increased interactions with the pig-handler, body/carcass lesions, occurrence of non-ambulatory and non-injured pigs, stressful experiences, and consequently lower meat quality [1,4]. Additionally, previous unpleasant experiences during on-farm handling reduce pigs’ motivation to interact with humans [5].

The group size plays an important role in pigs’ welfare and quality of handling [4]. Several studies have demonstrated the importance of using small groups to improve ease of handling and reduce stress of animals [4,6]. Handling pigs in smaller groups (five to six pigs vs. more than seven) reduced their heart rate and the time spent [1]. Indeed, large groups reduce the handler’s capacity of controlling the incidence of unwanted pig behaviors, such as turning around and stopping [1]. Similar results were found by Berry et al. [7] who reported disadvantages in the handling of larger groups (eight pigs vs. four), such as increased time spent in handling, and fatigued and dead pigs after handling. These disadvantages can be increased in situations when pigs present any of the following on-farm problems: abscesses, walking difficulties, caudophagia, arthritis, or hernias [4]. The increased physiological challenges caused by excessive interactions between the pig-handler due to pigs’ behaviors, such as turning around, overlapping each other, and stopping, usually more observed in larger groups during handling, can result in psychological stress and fatigue metabolic condition of pigs. Thus, pigs can have increased blood cortisol levels and enough muscle glycogen depletion which leads to higher values of initial muscle pH at slaughter and impaired pork quality traits [8,9,10]. However, a few other studies evaluated the effects of moving finishing pigs in different group sizes from farm to slaughter on animal welfare, carcasses, and pork quality traits [11].

Therefore, this experiment was conducted to evaluate the influence of moving finishing pigs in different group sizes on time spent to move pigs and ease of handling on the farm and its effects on blood stress parameters, carcasses, and meat quality of pigs at the slaughterhouse under commercial conditions.

## 2. Materials and Methods

All experimental procedures performed in this study were approved by the institutional animal care committee on the basis of the current guidelines of the Animal Research Ethics Board from the University of Goiás (protocol number N.079/16).

### 2.1. Animals, Loading Facilities, and On-Farm Handling of Pigs

The amount of 360 crossbred pigs (body weight of 105 ± 0.9 kg) from five commercial swine growing-finishing farms (similarly designed) were randomly selected, ear tagged (two days before the experiment started) and assigned to each treatment: groups of three, five, and ten pigs (four repetitions/treatment/farm). Each finishing farm used in this study housed about 450 pigs (± 25) per cycle (170 days; all-in/all-out system) in pens (14 ± 1 pigs/pen) on concrete floor at an average density of 1.15 m^2^/pig with a linear feeder and nipple drinkers. All farms were located in the South of Brazil. Before the handling to the slaughter, pigs were fasted for 12 h, as recommended by animal welfare literature [12]. At exit of the finishing pen, pigs were separated on the day of loading to slaughter. According to each treatment, pigs were moved by a trained loader using handling tools such as paddles and rattles [13] always between 23.00 h and 24.00 h. The standard on-farm facilities were similar, and the standard handling route consisted of moving through the exit of the finishing pen to the beginning of the loading area (typically a total of 50 m × 1 m) without corners. Hence, once pigs were selected and assigned to a moving group size, they were handled through the standard handling route in their group sizes on the farm and at the slaughterhouse. All farms had an adjustable loading ramp (11.0 ± 0.5 m long) according to the truck’s deck (15° and 21° slope for bottom and upper deck, respectively). Pigs were loaded within an average of 29.8 min (± 4.2 min) per truck.

### 2.2. Ease of Handling and Time Elapsed in Handling

Handling time was recorded between the limits set up from the pen gate (when crossed by the first pig’s nose) until the gate of the loading area (when crossed by the rear leg of the last pig) for all groups (four repetitions/treatment/farm). The ease of handling score was assigned by a trained observer based on a subjective scale ranging from extremely easy (score 1) to extremely difficult handling (score 5) similar to what has been previously described [14]. Briefly, this score was based on the direct observation of the frequency of the following behaviors: slips, falls, vocalizations, pig stopping, and interactions between handler-pigs. In this case, lower frequency of slips, falls, vocalizations, pig stopping, and contacts between handler and pigs meant lower scores (easier to handle).

### 2.3. Truck Design and Transport Conditions

The transport of pigs to the slaughterhouse was made using a double-decked truck (Triel-HT, Erechim, Brazil) with a fixed upper deck and a leaf-spring suspension system at a density of 230 kg/m^2^. A technical description of the truck is presented in [14,15]. The truck was driven straight to the slaughterhouse without stops.

### 2.4. Handling of Pigs at the Slaughterhouse

At the slaughterhouse, trucks were unloaded promptly at the arrival of the truck by a trained crew from the slaughterhouse using hand-held paddles. The slaughterhouse was equipped with an adjustable sloping metal ramp (5 m length, slope ≤ 15°) with anti-skid floor to allow the unloading of pigs.

Lairage pens with a density of 0.6 m^2^/100 kg were used at the slaughterhouse to allow pigs to rest (no social mixing; 5 m length × 4 m width × 1.5 m wall height) for 3 h. Pens had concrete floor and walls with solid metal entry/exit gates and one nipple drinker for fifteen pigs with a flow of 2 L/min. During the period of data collection, the ambient temperature at the slaughterhouse was controlled with a sprinkler and forced ventilation system (range of 15–21 °C).

At the end of lairage, pigs were conducted to the slaughter point in a very similar way to what was previously described on-farm using handling tools (paddles and rattles) according to each treatment. Pigs were mixed at the lairage but only between groups of the same treatment. To help ensure that all treatments received the same type of handling, pigs were moved through a standard handling course by the same trained handler. This standard handling route consisted of moving pigs through the exit from the lairage area to the beginning of the restraining equipment (±30 m for all treatments) with one corner of 90°. So, once the pigs were selected and assigned to a moving group size, they were handled through the standard routes in the same group sizes.

Pigs were head-only electrically stunned (700 V, 1.25 A, 5 s; Valhalla, Stork RMS b.v., Lichtenvoorde, Holland) before exsanguination in the horizontal position within 30 s. During the data collection of this study, the slaughterhouse operated at 280 pigs/h.

### 2.5. Physiological Measurements

Serum cortisol and plasma lactate were evaluated in blood samples (10 mL) collected in tubes (Vacuplast, Cral Artigos para Laboratório Ltd., São Paulo, Brazil) during horizontal exsanguination from a total of 180 pigs (60 pigs/treatment). For the plasma lactate analysis (2 mL), disposable tubes containing 3.0 mg of NaF and 6.0 mg of Na_2_EDTA solution were used.

After collection, the blood samples were centrifuged at 4 °C for 12 min at 1400× *g*. The plasma samples used for lactate concentration analysis were stored in identified Eppendorf tubes (1.5 mL) at −80 °C. Serum samples were kept at room temperature (~23 °C) for 1 h before being stored at 4 °C for 1 d to be processed. After that, they were centrifuged at 4 °C for 12 min at 1400× *g* to separate the supernatant, which was relocated into identified Eppendorf tubes (1.5 mL) and stored at −80 °C for further analysis. Blood lactate and cortisol levels were assessed using commercially available kits (Lactat PAP Enzyme Farbtest, Rolf Greiner Biochemica, Flacht, Germany and Coat-A-Count Cortisol Kit, Diagnostic Products Corporation, Los Angeles, CA, USA, respectively) with a microplate reader. The CV was 10.0% and 32.2% for lactate (range 1.71–14.16 mmol·L^−1^) and cortisol (range 6.34–12.98 μg/dL) concentrations, respectively.

### 2.6. Carcass Handling and Skin Lesion Assessment

Carcasses were eviscerated, split, and chilled (1–4 °C for 24 h) following the standard commercial practices adopted in the slaughterhouse. Carcass evaluation (*n* = 360) consisted of visual assessment according to lesions shapes and sizes as described by [16,17] and classified as mounting-type lesions (score 1 = fewer than 5 lesions; 2 = 6 to 10 lesions; and 3 = greater than 10 lesions) and fighting-type lesions (1 = fewer than 10 lesions; 2 = 11 to 20 lesions; and 3 = greater than 20 lesions, comma shaped with 5 to 10 cm in length).

### 2.7. Meat Quality

The same 360 carcasses were used for meat quality assessments. Both muscles Longissimus thoracis (LT; between the 13th and 14th rib) and Semimembranosus (SM) were used to evaluate pH (HI 8314 model, Hanna Instruments, São Paulo, Brazil) at 45 min (pH_i_) and 24 h (pH_u_) post-mortem (in the LT and SM). Objective and subjective color (Minolta L*,a*,b* using CR-400; Minolta Camera Ltd., Osaka, Japan; and NPPC Pork Quality Standards from National Pork Producers Council, 1999, respectively) and drip loss were evaluated in the same samples previously used at 24 h post-mortem in the LT muscle according to the modified EZ-drip loss method [18].

The cooking losses were evaluated in four LT muscle samples of approximately 150 g. Samples were vacuum-packed in individual heat resistant plastic bags (nylon polyethylene bag 16 × 30 × 0.1 cm), 10 μm thick and sealed with a vacuum of 0.8 bar with a double seal by a DZ-4000 vacuum packer machine (Cetro Solutions in packing, Taiwan, China). All plastic bags were cooked in water bath at 80 °C for 1 h at 48 h post-mortem. Then, the residual surface moisture was removed using an absorbent paper and the samples were reweighed when the internal temperature reached 20–25 °C. The weight loss was calculated according to [19].

The same cooked LT muscle samples were then cooled at 0 °C for 12 h, cut into five rectangular cores (1 × 1 × 2 cm) parallel to the longitudinal orientation of the muscle fibers, cooked, and used for the determination of Warner–Bratzler shear force using a Warner–Bratzler device attached to a TAXT2i Texture Analyzer (Stable Micro Systems, Surrey, UK). The meat quality classification was calculated according to the quality criteria set for this study [14,15].

### 2.8. Statistical Analysis

Data was analyzed as a factorial of 3 × 5 (3 treatments × 5 farms) to check the effects of treatments by analysis of variance in ANOVA. Values of serum cortisol and plasma lactate concentration were log-transformed (Ln) for data normalization before analysis. Frequencies of lesions were transformed as the square root of (x + 1) and are presented in the tables as back-transformed means. The model included effects of block (farm), treatments, interaction between treatments and farms (block), and error (corresponding to randomized variation in the observations on the day at the farm), supposedly homoscedastic, independent, and normally distributed. Variance analysis using GLM SAS (2012) was applied to study the effects of treatments using the group as experimental unit for the analysis of behavioral data and the individual as the experimental unit for the analysis of physiological and meat quality data. The Fisher exact test was applied to evaluate the independence of scores’ profiles for the treatments studied. The likelihood ratio and chi-square tests were used to compare the skin lesion-type categories. The tests were performed using the FREQ procedure of SAS (2012) with Student’s t test protected by the significance of the F test for mean comparison. A probability level of *p* < 0.05 was chosen as the limit for statistical significance in all tests, and probability levels of *p* < 0.10 were considered as a tendency. There was no significant effect and interaction of farm, block, and farm, or block in any of the studied variables (*p* > 0.10).

## 3. Results and Discussion

### 3.1. Ease of Handling and Time Elapsed During the Practice

In agreement with the literature [1,6,7], smaller groups were easier to handle than bigger ones (Table 1). Based on the handling scores, groups with fewer than 10 pigs were reasonably easier (*p* < 0.05) to handle due to the fewer occurrence of animals turning back and stopping. As the group size increased, the ease of handling decreased. Indeed, the score 1 (easiest) was only observed in groups with three pigs, which did not have a score greater than 4.0 (more difficult handling). The score 5 (hardest) was only observed in the groups of 10 pigs, which had a minimum score of 3.0. The group size compared in this study did not increase the time required to move the pigs (2.60 ± 1.05 vs. 2.42 ± 0.82 vs. 2.55 ± 1.23 min/per replicate for groups of 3, 5, and 10 pigs, respectively; *p* > 0.10). Lewis and McGlone [1] reported that the time required for loading a truck decreased from 1 to 5 pigs per group, and groups larger than 5 pigs (6–10) required similar times. Dalla Costa et al. [14] reported greater times for loading when ease of handling was more difficult. Therefore, no time savings were found in both studies when bigger group sizes (>5 pigs) were handled.

### 3.2. Physiological Response

Only serum cortisol levels tended to be affected by treatments (*p* < 0.07; Table 2). Pigs handled in groups of three and ten pigs tended to have higher (*p* < 0.70) serum cortisol levels than pigs handled in groups of five pigs. Increased serum cortisol levels at exsanguination indicate an incomplete recovery from the psychological stress of handling pigs in too small or large groups. A possible explanation for this finding is the greater handling score observed in the larger group, mainly due to the higher frequency of turning around and stopping behaviors. Lewis and McGlone [1] state that when the threshold of seven pigs per group is exceeded, the handler loses control over each individual pig more easily than with smaller groups, and pigs begin to show undesirable behaviors (such as turn back, turn on another, and stopping) more frequently, resulting in more difficult handling. As previously reported [15], the high CV values found in this study are commonly found and acceptable for these kinds of analyses. This variance reflects the individual effect on these variables and its contribution to the variance. The study was conducted under commercial conditions, and physiological responses evaluated the influence of handling group size and the sum of effects cause by the preslaughter procedures. Since the effects of loading and unloading were very similar between treatments, there were no strong effects of these phases on the response. The moment of blood sample collections represents a sum of the effects (loading, transport, unloading, treatments, and stunning procedures). However, the experimental design was planned to make the treatment groups receive the same conditions and so have the same random influences. Thus, the differences would represent only the effects of the handling group size.

### 3.3. Skin and Carcass Lesions

The group size significantly affected (*p* < 0.05) carcass lesions, especially over the loin, caused by fighting and handling. As group size increased, the loin and total of carcass lesions caused by fighting and handling tended to increase (Table 3). However, groups of 10 pigs had fewer injuries caused by mounting, presumably because those pigs had fewer space to perform this type of behavior. Other work has also shown that handling pigs in larger group sizes (>7) may increase carcass lesions due to pigs mounting/stepping on each other and fighting [1]. It is likely that higher handling scores (more difficult handling) involve more interactions between pigs and handlers and lead to poorer handling, resulting in more carcass lesions. Based on that, the increased number of carcass lesions in larger groups is likely due to the higher occurrence of unwanted pig behaviors and interactions between animals and handlers, and this may be avoided by handling in smaller group sizes. Independent of the cause, the group size adopted during handling also involves welfare problems in the form of pain from bruises, injuries, and carcass lesions.

### 3.4. Meat Quality

Although the pork quality classification was not affected (*p* > 0.10), some pork quality traits were significantly influenced by different group sizes. Pigs handled in the groups of three and ten animals had a higher (*p* < 0.05) pH_u_ and initial temperature in LT and higher (*p* < 0.05) pH_u_ and initial temperature as well as lower drip loss in Semimembranosus (Table 4). These effects on pork quality may be partly explained by the higher frequencies of handling difficulty and fighting behaviors in the larger groups (10 pigs) as previously discussed. When handled in groups of three animals, pigs tended to have a higher frequency of mounting behavior and were more stressed in terms of the serum cortisol. Although muscle temperatures tended to be higher in the groups of three and ten pigs, they were not metabolically acidotic at slaughter. Taken together, these results indicate that the pigs in the three and ten pig group size treatments had been more metabolically stressed by the time they reached the slaughter point. However, these conditions of stress were not such to cause either a metabolic acidosis or PSE (pale, soft and exudative) or DFD (dark, firm and dry) meat. An implication from this study is that in situations where pigs are prone to hyperthermia immediately before slaughter, moving them in groups of 5 animals may improve meat quality through limiting the increase in muscle temperature.

## 4. Conclusions

Moving pigs in groups of five animals minimizes their stress and improves animal welfare, while maintaining a reasonable quality of handling during on-farm practices. These results will help stimulate the use and adoption of improved handling methods and strategies to ensure better animal welfare, especially when moving pigs before slaughter.

## Figures and Tables

**Table 1:** Effect of group size on frequency of handling score (%) on pigs ^1^.

Group Size (Pigs)	Scores								
	1.0	1.5	2.0	2.5	3.0	3.5	4.0	4.5	5.0
3 ^a^	15	5	40	20	15	0	5	0	0
5 ^b^	0	0	15	20	25	30	10	0	0
10 ^c^	0	0	0	0	10	50	25	10	5

^1^ Twenty groups/treatment, ^a,b,c^ Frequencies profiles followed by a different superscript differ by Fisher’s exact test (*p* ≤ 0.05).

**Table 2:** Effect of moving different group sizes on blood stress indicators^1^ in pigs.

Parameters	Group Size (Pigs)			
	3	5	10	*p*-Value
Cortisol (μg/dL)	7.20 ± 0.71 ^a^	5.83 ± 0.50 ^b^	6.47 ± 0.45 ^a,b^	0.070
Lactate (mmol·L^−1^)	11.07 ± 0.29	11.41 ± 0.32	11.63 ± 0.20	0.370

^1^ Mean values and standard error of mean of serum cortisol and plasma lactate. ^a,b^ Means lacking a common superscript differ (*p* > 0.05) in the Tukey test. *p* < 0.10 was considered to be a tendency.

**Table 3:** Number of carcass lesions (mean ± SEM) in pigs handled in different group sizes.

Site of Carcass	Group Size (Pigs)			
	3	5	10	*p*-Value
Fighting lesions				
Loin	1.44 ± 0.24 ^b^	1.44 ± 0.17 ^b^	2.14 ± 0.31 ^a^	0.047
Thigh	4.67 ± 0.70	4.35 ± 0.53	5.32 ± 0.54	0.475
Ham	1.09 ± 0.19	1.12 ± 0.14	1.31 ± 0.21	0.629
Total	7.21 ± 0.93	6.91 ± 0.62	8.77 ± 0.81	0.203
Mounting lesions				
Loin	1.64 ± 0.15 ^a^	1.61 ± 0.11 ^a^	1.17 ± 0.14 ^b^	0.012
Thigh	0.06 ± 0.05	0.00 ± 0.00	0.01 ± 0.01	0.162
Ham	1.46 ± 0.10	1.44 ± 0.12	1.46 ± 0.08	0.985
Total	3.16 ± 0.18 ^a^	3.05 ± 0.20 ^a^	2.64 ± 0.17 ^b^	0.038
Handling lesions				
Loin	1.96 ± 0.25 ^b^	2.68 ± 0.26 ^a^	2.73 ± 0.32 ^a^	0.047
Thigh	0.26 ± 0.07	0.53 ± 0.15	0.27 ± 0.13	0.219
Ham	0.71 ± 0.09	0.73 ± 0.09	0.83 ± 0.13	0.661
Total	2.93 ± 0.36	3.94 ± 0.44	3.83 ± 0.49	0.108

^a,b^ Means lacking a common superscript indicate significant difference (*p* < 0.05). *p* < 0.10 was considered to be a tendency.

**Table 4:** Effects of moving different group sizes of pigs on meat quality traits^1^.

Variable ^2^	Group Size (Pigs)			
	3	5	10	*p*
LT muscle (Loin)				
pH_i_	6.29 ± 0.05	6.27 ± 0.03	6.27 ± 0.04	0.858
T_i_ (°C)	31.90 ± 0.40 ^a^	31.25 ± 0.55 ^b^	31.63 ± 0.36 ^a^	0.004
pH_u_	5.61 ± 0.02	5.57 ± 0.03	5.63 ± 0.03	0.058
T_u_ (°C)	4.52 ± 0.27	4.33 ± 0.21	4.40 ± 0.22	0.454
Driploss (%)	3.82 ± 0.26	3.55 ± 0.18	3.59 ± 0.25	0.504
L *	45.20 ± 0.46	45.04 ± 0.25	45.00 ± 0.30	0.880
a *	6.18 ± 0.23	6.34 ± 0.21	6.20 ± 0.15	0.656
b *	−0.08 ± 0.24	0.29 ± 0.16	0.11 ± 0.30	0.079
SS (N)	57,86 ± 0.28	60,31 ± 0.27	61.88 ± 0.25	0.412
WLC (%)	30.85 ± 0.23	31.27 ± 0.24	31.16 ± 0.19	0.294
SM muscle (Ham)				
pH_i_	6.32 ± 0.04	6.30 ± 0.03	6.29 ± 0.04	0.583
T_i_ (°C)	32.27 ± 0.40 ^a^	31.57 ± 0.54 ^b^	31.89 ± 0.38 ^b^	0.001
pH_u_	5.64 ± 0.02 ^a^	5.58 ± 0.04 ^b^	5.65 ± 0.02 ^a^	0.045
T_u_ (°C)	4.40 ± 0.25	4.26 ± 0.21	4.36 ± 0.22	0.517
Driploss (%)	2.21 ± 0.22 ^b^	2.78 ± 0.19 ^a^	2.53 ± 0.21 ^b^	0.032
L *	45.43 ± 0.46	44.92 ± 0.48	45.25 ± 0.42	0.402
a *	6.51 ± 0.31	6.72 ± 0.21	6.61 ± 0.16	0.410
b *	0.01 ± 0.28	0.25 ± 0.19	0.21 ± 0.25	0.284

^1^ Mean values and standard error of pH_i_ = pH 45 min post mortem; pH_u_ = pH 24 h post mortem (pH_u_); T_1_ = muscle temperature at 45 min post mortem; T_u_: muscle temperature at 24 h post mortem; L = luminosity; a = red color; b = yellow color; N = Newtons; WLC = water loss during cooking; SS = shear force. ^2^ LT muscle = Longissimus thoracis; SM = Semimembranosus. ^a,b^ Means lacking a common superscript indicate significant difference (*p* < 0.05) by Student’s t test, protected by the significance of the F test. *p* < 0.10 was considered to be a tendency.
